# Testicular Germ Cell Tumor Composed of Seminoma and Teratoma Metastasizing as Choriocarcinoma to the Lung Successfully Treated With Salvage Chemotherapy and Bone-Marrow Transplant: A Case Report

**DOI:** 10.7759/cureus.22885

**Published:** 2022-03-06

**Authors:** Soroush Shahrokh, Daniel Tran, Barbara Lines, Murtaza N Bhuriwala

**Affiliations:** 1 Graduate Medical Education, HCA Houston Healthcare Kingwood Medical Center/University of Houston College of Medicine, Kingwood, USA; 2 Internal Medicine Residency Program/Internal Medicine, HCA Houston Healthcare Kingwood Medical Center/University of Houston College of Medicine, Kingwood, USA; 3 Pathology, HCA Houston Healthcare Kingwood Medical Center, Kingwood, USA; 4 Hematology and Medical Oncology, HCA Houston Healthcare Kingwood Medical Center, Kingwood, USA

**Keywords:** choriocarcinoma, teratoma, seminoma, mixed germ cell tumor, testicular cancer metastasis, testicular germ cell tumor, testicular tumor, testicular cancer

## Abstract

A 25-year-old male presented to our hospital with two months of progressively worsening left arm swelling, intermittent left-sided chest pressure, and a painless right testicular mass. CT of the chest, abdomen, and pelvis revealed a large mediastinal mass, multiple lung nodules, and several large right testicular nodules. The patient underwent a CT-guided biopsy of his right lung nodule, followed by a radical right inguinal orchiectomy. The testicular biopsy revealed a mixed germ cell tumor (GCT) consisting of 97% seminoma and 3% teratoma, while the lung biopsy revealed metastatic choriocarcinoma. The patient was treated with four cycles of bleomycin, etoposide, and platinum (BEP) and showed a great clinical response, with only residual disease in his retroperitoneal lymph nodes. He was referred for retroperitoneal lymph node dissection (RPLND); however, there was a delay of several months, which led to the recurrence of his disease. He received four cycles of paclitaxel, ifosfamide, and cisplatin and showed a moderate response. He later received salvage chemotherapy with high-dose carboplatin and etoposide and underwent bone-marrow transplant, leading to complete clinical response and eradication of his disease.

There are different subtypes of testicular GCTs, each with distinct pathogenesis, treatment modality, and prognosis. In this report, we discuss the case of a patient who presented with a mixed GCT consisting of seminoma and teratoma in his testicle, which had metastasized as choriocarcinoma to his lung and mediastinum. This report elucidates the potential for testicular GCTs to metastasize as a pathologically different cancer compared to the primary tumor. This phenomenon has significant clinical ramifications, as it can considerably alter a patient’s treatment and prognostic outcomes.

## Introduction

Testicular cancer is a rare malignancy, accounting for only 1% of all malignant tumors in men [1]. However, it is the most common cancer in men aged 15-40 years, with its global incidence steadily rising [2-4]. About 95% of testicular cancers are germ cell tumors (GCTs), which are broadly divided into seminomatous and non-seminomatous GCTs (NSGCTs) [5]. NSGCTs include teratomas, yolk sac tumors, embryonal carcinomas, choriocarcinomas, and mixed tumors [5]. Seminomatous GCTs have an excellent prognosis with a five-year survival rate of approximately 90%, while NSGCTs are less sensitive to chemoradiation and have a less favorable outcome [2-4]. The mainstay treatment modalities for testicular cancer include orchiectomy, retroperitoneal lymph node dissection (RPLND), and chemoradiation [6,7].

In this report, we describe a rare case of a patient with mixed testicular GCT composed of seminoma and teratoma, which had transformed into choriocarcinoma after metastasizing to his lungs and the mediastinum. Current guidelines recommend orchiectomy alone for the diagnosis and initial treatment of testicular GCTs of stage III or higher [6,7]. While RPLND is recommended for a subset of patients, obtaining a biopsy of the metastatic lesions is generally not recommended [7]. However, because of the potential of the testicular GCTs to transform into a more aggressive neoplasm upon metastasis, as demonstrated in our patient, it may be prudent for clinicians to obtain biopsies from both the primary and metastatic lesions.

## Case presentation

**Figure 1:**
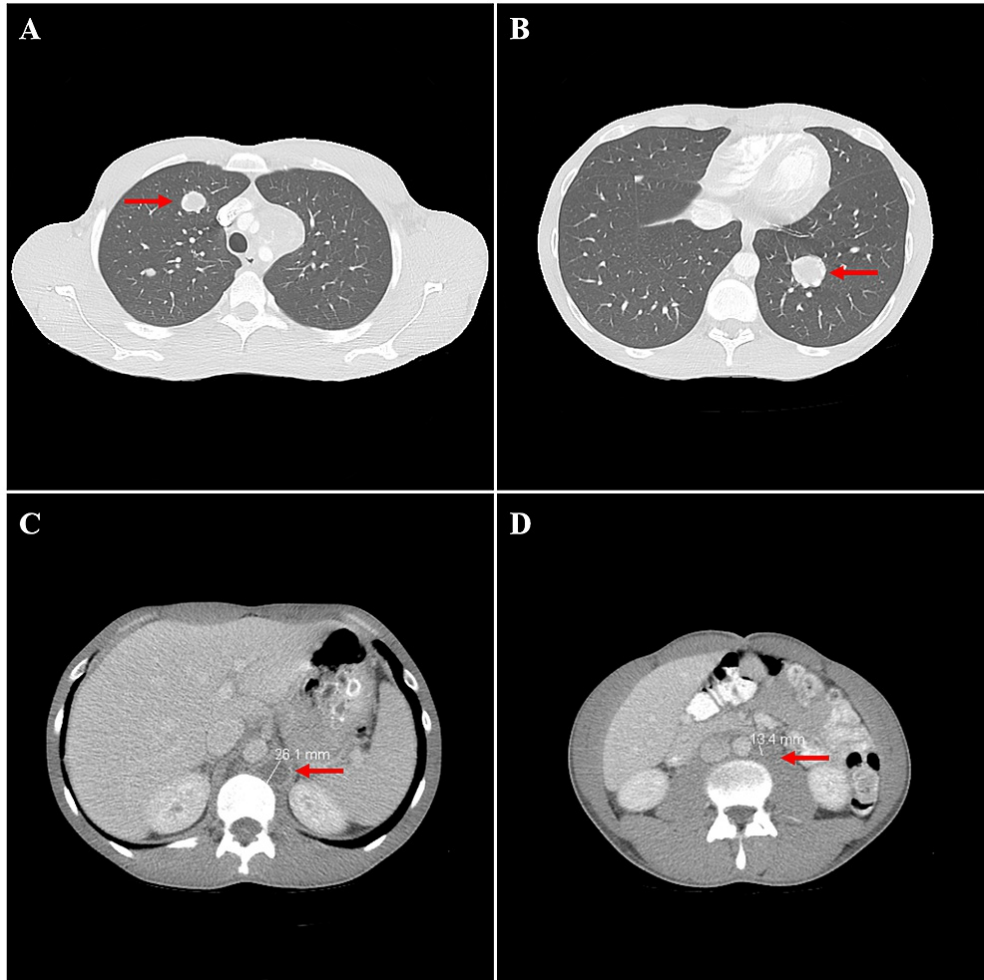
CT chest during patient’s hospital admission showed a left lower lobe lung mass measuring 2.8 cm (A) (red arrow) and a large heterogeneously enhancing mass in the anterior upper mediastinum consistent with metastatic disease (B) (red arrow). CT abdomen and pelvis showed left retrocrural adenopathy measuring up to 2.8 cm in the upper abdomen (C) (red arrow) and para-aortic lymphadenopathy with lymph nodes measuring up to 1.7 cm (D) (red arrow) CT: computed tomography

A 25-year-old Caucasian male with no past medical history presented to the emergency department with two months of progressively worsening left arm swelling and pain along with intermittent left-sided chest pressure. He had also noticed a small mass in his right testicle three months ago and had an unintentional 10-pound weight loss over the same period. On physical exam, he had a painless mass in the upper pole of his right testicle and firm, non-tender inguinal lymphadenopathy. His left arm was edematous and mildly colder than his right arm. His electrocardiogram and cardiac enzymes were normal, but his chest X-ray revealed a 2.6-cm density projecting over the middle and lower lobes of his left lung. His chest CT angiogram revealed an 8.3-cm heterogeneous enhancing mass in his anterior mediastinum, a 2.8-cm mass in the lower lobe of his left lung, and several scattered nodules throughout his bilateral lungs (Figure 1). CT of the abdomen and pelvis revealed left-sided retroperitoneal and retrocrural adenopathy, and the scrotal ultrasound showed multiple solid, sharply defined, and mildly vascular hypoechoic nodules in his right testicle. The patient’s initial tumor markers showed a markedly elevated β-human chorionic gonadotropin (β-hCG) of 68,180 mIU/mL (normal range: <2 mIU/mL) and lactate dehydrogenase (LDH) of 931 U/L (normal range: <280 U/L) but a normal α-fetoprotein (AFP) of 3.3 ng/mL (normal range: <20 ng/mL). Urology and medical oncology services were consulted, and they gave a preliminary diagnosis of lymphoma versus metastatic testicular cancer. The patient underwent a CT-guided biopsy of his right lung nodule, followed by radical right inguinal orchiectomy. Both specimens were sent for pathologic evaluation.

**Figure 2:**
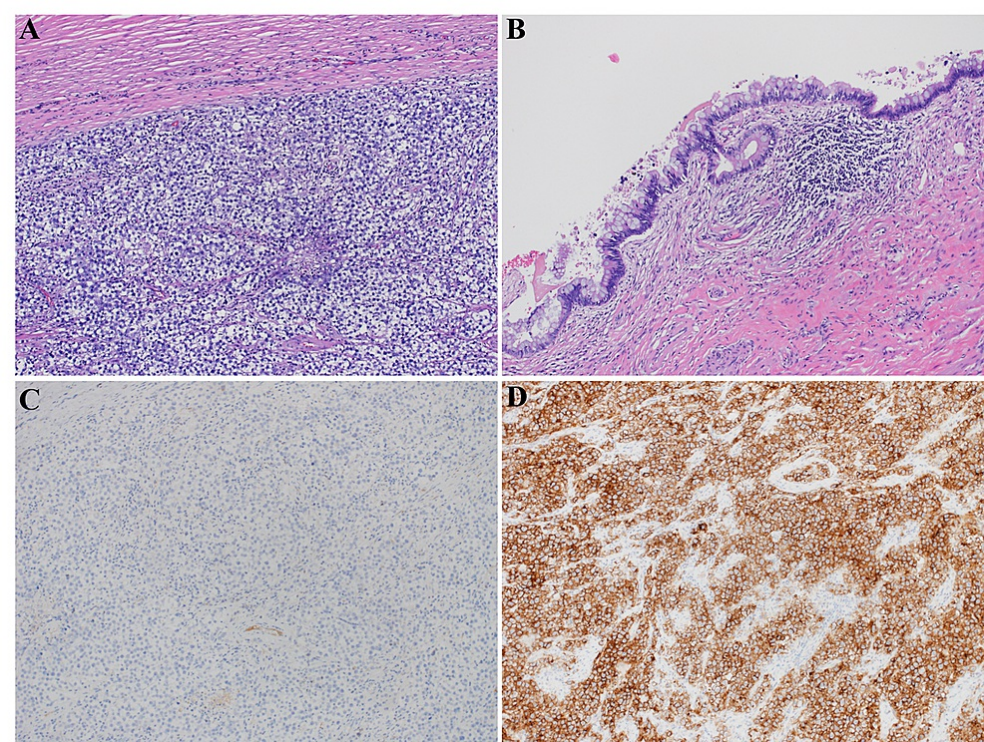
Hematoxylin and eosin (H&E) stain of the patient’s right testis biopsy showing the presence of seminoma (A) and teratoma (B). (C) Positive D2-40 immunohistochemical staining consistent with the presence of seminoma. (D) Negative β-hCG immunohistochemical staining consistent with the absence of choriocarcinoma β-hCG: β-human chorionic gonadotropin

**Figure 3:**
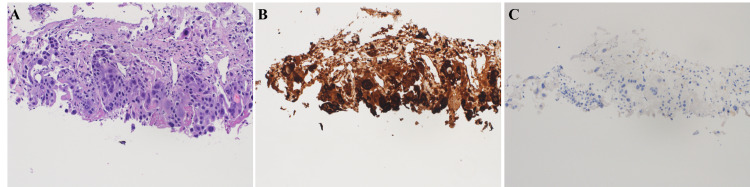
A. High-power hematoxylin and eosin stain of the patient’s lung nodule revealing metastatic choriocarcinoma. B. Positive β-hCG immunohistochemical staining consistent with choriocarcinoma. C. Negative OCT-4 immunohistochemical staining consistent with the absence of seminoma β-hCG: β-human chorionic gonadotropin

Microscopic examination and immunohistochemical (IHC) study of the right testicle showed a mixed testicular GCT composed of 97% seminoma and 3% teratoma (Figures 2A, 2B). However, the pathologic examination of the lung nodule revealed metastatic choriocarcinoma, with a similar morphological appearance as the right testicular GCTs (Figures 3A, 3B, 3C). Extensive sampling of the testicular tumor with accompanying IHC studies failed to demonstrate the presence of any choriocarcinoma component in the right testicle (Figures 2C, 2D). The patient’s repeat tumor markers one day after orchiectomy revealed a persistently elevated β-hCG of 69,642 mIU/mL and LDH of 1,189 U/L with a normal AFP of 4.2 ng/mL.

**Figure 4:**
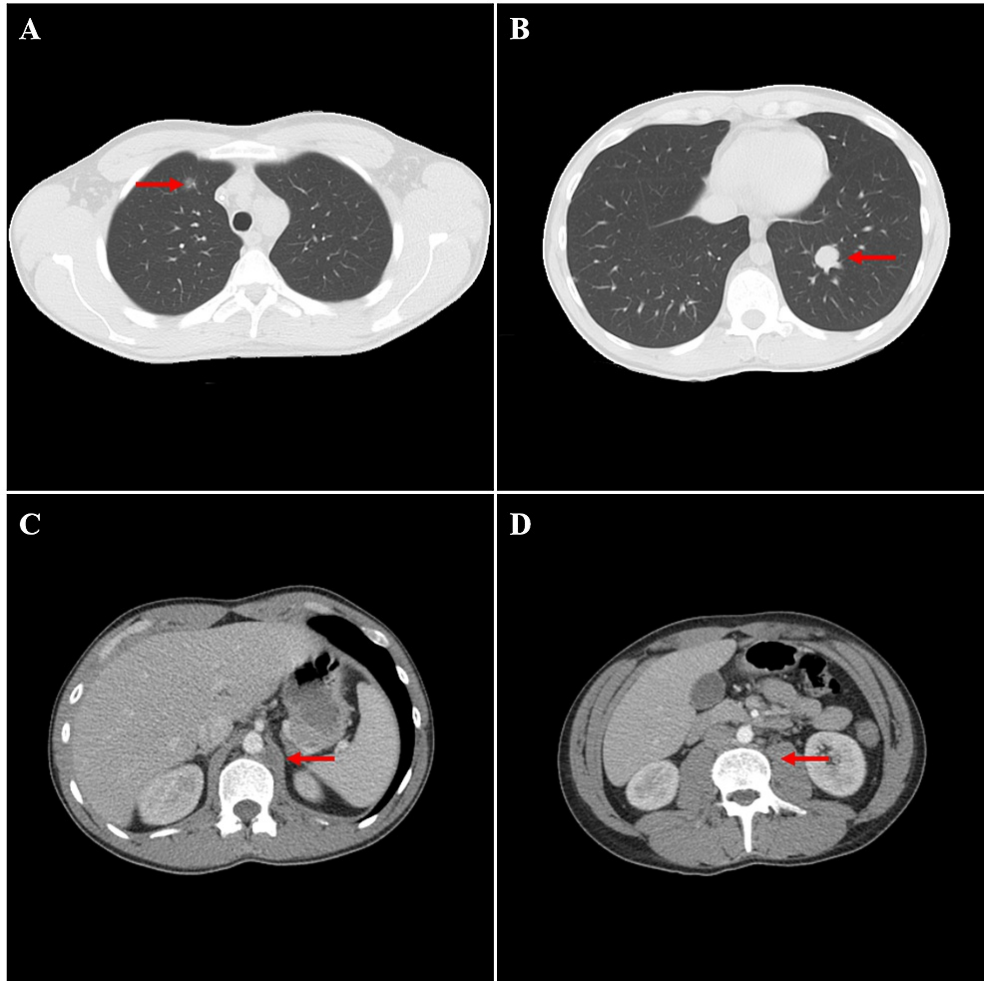
CT chest after the completion of four cycles of BEP chemotherapy showed a positive response to treatment with significant shrinkage of the left lower lobe lung mass (A) (red arrow) and a significant decrease in the size of the right anterior mediastinal mass, consistent with a positive response to treatment (B) (red arrow). CT abdomen and pelvis showed a marked shrinking of the left retroperitoneal lymphadenopathy (C) (red arrow) and disappearance of the para-aortic lymphadenopathy (D) (red arrow) CT: computed tomography; BEP: bleomycin, etoposide, and platinum

The patient was treated with systemic chemotherapy with four cycles of bleomycin, etoposide, and platinum (BEP). After completing the chemotherapy course, the patient’s chest, abdomen, and pelvis CT scans revealed a significant clinical response, with only residual metastatic disease remaining in his retroperitoneal lymph nodes (Figure 4). Also, the patient’s serum β-hCG had dropped to less than 20 mIU/mL. He was referred to a tertiary care cancer center for RPLND; however, there was a three-month delay due to financial constraints, which led to the recurrence of his disease. He received four cycles of chemotherapy with the paclitaxel, ifosfamide, and cisplatin (TIP) regimen. He initially showed a moderate clinical response after three treatment cycles; however, he subsequently developed chemo-resistance and had a significant disease progression. The patient was later treated with salvage chemotherapy with high-dose carboplatin and etoposide along with bone-marrow transplant, which led to complete disease eradication.

## Discussion

Testicular GCTs constitute approximately 95% of all testicular malignancies and are broadly classified as seminoma and non-seminoma subtypes [5]. This classification is used for treatment purposes and determining prognostic outcomes. Seminomas are highly sensitive to chemoradiation and have an excellent prognosis, with cure rates of nearly 100% in patients with stage I seminoma [8-10]. However, NSGCTs, especially choriocarcinomas, are much more aggressive and have a significantly higher rate of resistance to chemoradiation, leading to significantly less favorable outcomes [8,9].

The initial diagnostic imaging of testicular cancers includes a scrotal ultrasound. If ultrasound findings raise suspicion for malignancy, whole-body imaging, including CT of the abdomen and pelvis, chest, and brain MRI, is performed to detect metastatic disease [8]. Additionally, the patient’s serum tumor markers for testicular GCTs (i.e., β-hCG, AFP, and LDH) are measured periodically to determine the patient’s prognosis and therapeutic response [8,9]. Elevated serum AFP is seen in GCTs that contain embryonic yolk sac cells, including embryonal carcinomas, yolk sac tumors, or teratomas [10-12]. Neither seminoma nor choriocarcinoma contains embryonic yolk sac cells, and neither is associated with an increase in serum AFP. β-hCG is a glycoprotein secreted by the syncytiotrophoblasts, the neoplastic components of both seminomas and choriocarcinomas [10-12]. Although both seminomas and choriocarcinomas secrete β-hCG, seminomas are typically associated with β-hCG <1,000 mIU/mL, while choriocarcinoma is associated with β-hCG >5,000 mIU/mL [12]. Our patient had a normal serum AFP level of 4.2 ng/mL; however, his serum β-hCG levels were markedly elevated at 68,180 mIU/mL, which continued to increase even after his orchiectomy. Although a few cases of bulky pure seminomas causing β-hCG >10,000 mIU/mL have been reported, it is generally a rare phenomenon [13]. This persistent elevation of β-hCG indicated that our patient likely had GCT components other than pure seminoma.

Previous studies have shown that approximately 30-50% of primary testicular GCTs consist of more than one cell component [14]. This phenomenon is widespread in NSGCTs, more than 60% of which are composed of more than one neoplastic cell type [14]. This phenomenon is most likely due to the common embryonic origin of all GCTs from primitive, pluripotent germ cells, which can transform into different malignant cell types [9]. Furthermore, studies have frequently shown a discordance between the histologic composition of the primary testicular GCT and its corresponding metastasis [14-17]. Multiple theories have been proposed to explain the cause of this phenomenon. Tarrant et al. proposed that this discrepancy may be caused by the conversion of primary testicular GCTs from a less mature embryonic cell type into its more mature neoplastic counterpart [14]. According to this theory, GCTs originating from embryonic carcinoma would only metastasize as their more mature histological cell type; this would cause the conversion of teratoma, the primitive germ cell during embryogenesis, to choriocarcinoma, which is its more mature and well-differentiated counterpart [14]. Tarrant et al. further speculated that since totipotent germ cells are the precursors of all GCTs, their forward and backward differentiation can lead to the difference in the histopathology of the primary versus metastatic cancer cells [14]. Other studies have suggested that this discordance can be caused by the inherent resistance of some GCT components to chemotherapy, leading to their survival and selective proliferation [14,18].

Our patient’s concomitant testicular seminoma and metastatic choriocarcinoma support the theory of backward conversion of seminoma into teratoma, which subsequently metastasized to his lungs. After metastasizing, the teratoma had a forward conversion into its more mature neoplastic counterpart, choriocarcinoma. Our patient had not undergone any chemotherapy before, which renders the possibility of selective proliferation of chemo-resistant malignant cells inapplicable.

## Conclusions

Testicular GCTs are the most commonly diagnosed cancer in young men. Due to their heterogeneity and pluripotent nature, testicular GCTs have the potential to transform into a more aggressive neoplasm after metastasizing. Therefore, it may be prudent for clinicians to obtain biopsies from primary and metastatic lesions. This practice would increase the likelihood of detecting metastatic tumor components not in the primary tumor site, with significant ramifications for the patient’s diagnosis, treatment, and expected prognosis. In this report, we elucidated the potential of testicular GCTs to transform into a more aggressive tumor after metastasizing.
